# Cross-reactivity of a rice NLR immune receptor to distinct effectors from the rice blast pathogen *Magnaporthe oryzae* provides partial disease resistance

**DOI:** 10.1074/jbc.RA119.007730

**Published:** 2019-07-11

**Authors:** Freya A. Varden, Hiromasa Saitoh, Kae Yoshino, Marina Franceschetti, Sophien Kamoun, Ryohei Terauchi, Mark J. Banfield

**Affiliations:** ‡ Department of Biological Chemistry, John Innes Centre, Norwich Research Park, NR4 7UH Norwich, United Kingdom; § Laboratory of Plant Symbiotic and Parasitic Microbes, Department of Molecular Microbiology, Faculty of Life Sciences, Tokyo University of Agriculture, Tokyo 156-8502, Japan; ¶ The Sainsbury Laboratory, University of East Anglia, Norwich Research Park, NR4 7UH Norwich, United Kingdom; ‖ Division of Genomics and Breeding, Iwate Biotechnology Research Center, Iwate 024-0003, Japan; ** Laboratory of Crop Evolution, Graduate School of Agriculture, Kyoto University, Kyoto 606-8501, Japan

**Keywords:** plant defense, protein structure, plant biochemistry, host-pathogen interaction, Nod-like receptor (NLR), effector, plant immunity, rice, rice blast disease

## Abstract

Unconventional integrated domains in plant intracellular immune receptors of the nucleotide-binding leucine-rich repeat (NLRs) type can directly bind translocated effector proteins from pathogens and thereby initiate an immune response. The rice (*Oryza sativa*) immune receptor pairs Pik-1/Pik-2 and RGA5/RGA4 both use integrated heavy metal-associated (HMA) domains to bind the effectors AVR–Pik and AVR–Pia, respectively, from the rice blast fungal pathogen *Magnaporthe oryzae*. These effectors both belong to the MAX effector family and share a core structural fold, despite being divergent in sequence. How integrated domains in NLRs maintain specificity of effector recognition, even of structurally similar effectors, has implications for understanding plant immune receptor evolution and function. Here, using plant cell death and pathogenicity assays and protein–protein interaction analyses, we show that the rice NLR pair Pikp-1/Pikp-2 triggers an immune response leading to partial disease resistance toward the “mis-matched” effector AVR–Pia *in planta* and that the Pikp–HMA domain binds AVR–Pia *in vitro*. We observed that the HMA domain from another Pik-1 allele, Pikm, cannot bind AVR–Pia, and it does not trigger a plant response. The crystal structure of Pikp–HMA bound to AVR–Pia at 1.9 Å resolution revealed a binding interface different from those formed with AVR–Pik effectors, suggesting plasticity in integrated domain-effector interactions. The results of our work indicate that a single NLR immune receptor can bait multiple pathogen effectors via an integrated domain, insights that may enable engineering plant immune receptors with extended disease resistance profiles.

## Introduction

When plants encounter biotic stresses, they respond rapidly to defend themselves against attack. Microbial pathogens translocate effector proteins inside host cells to undermine plant immunity and promote pathogen growth and proliferation (1). To detect these effectors, plants have developed intracellular immune receptors, many of which are of the nucleotide-binding leucine-rich repeat (NLR)^2^ class (2). The hallmark feature of NLR-mediated immunity is the hypersensitive response, a programmed cell death around the site of infection that helps to isolate and halt the spread of the pathogen (3).

NLRs recognize effector proteins via different mechanisms, including by direct or indirect binding (4, 5). Some NLRs function in pairs, with one receptor responsible for recognizing the effector (referred to as the sensor), and one responsible for translating the recognition into a signaling response (the helper) (6). One mechanism to evolve direct binding has been for NLRs to integrate an unconventional domain into the protein architecture (7, 8), with this domain thought to be derived from the virulence-associated host target of the effector. Once integrated, these domains may adapt to recognize effectors (and different effector alleles). Their widespread distribution in NLRs from diverse plant species suggests this is an ancient mechanism for evolving effector recognition (9, 10).

Two paired rice NLR immune receptors are known that contain an integrated heavy metal-associated (HMA) domain, Pik-1/Pik-2 and RGA5/RGA4. In Pik, this domain is integrated between the coiled-coil and nucleotide-binding (NB-ARC) domains of Pik-1 (11, 12), but in the RGA pair the HMA domain is found at the C terminus of RGA5 (13). Both these pairs of immune receptors recognize effectors from the blast fungus *Magnaporthe oryzae*, a global threat to rice production causing loss of up to a third of the total annual harvest of this crop (14–16).

*M. oryzae* secretes a large repertoire of effector proteins, and many of these, including the structurally characterized AVR–Pizt, AVR–Pia, AVR–Pik, AVR1–CO39, and AVR–Pib (11, 17–19), share a conserved structure comprising a six-stranded β-sandwich known as the MAX (*Magnaporthe* Avrs and ToxB-like) fold (18, 20). Therefore, despite being sequence-unrelated, these effectors are all similar in overall shape.

The Pik-1/Pik-2 NLR pair recognizes the *M. oryzae* effector AVR–Pik (21), and both the NLRs and effectors are found as allelic series in natural populations (22). Direct interaction between the Pik–HMA domain and AVR–Pik is required for triggering an immune response to the effector (11). At the sequence level, the allelic Pikp (23) and Pikm (24) pair differs mainly in their polymorphic HMA domains (12), and this underpins different recognition specificities for different AVR–Pik alleles; Pikp is only able to recognize the effector variant AVR–PikD, whereas Pikm can recognize AVR–PikD and other additional AVR–Pik variants. The AVR–PikC effector variant is currently unrecognized by any Pik NLR (22).

The RGA5/RGA4 NLR pair responds to the *M. oryzae* effectors AVR–Pia (25) and AVR1–CO39 (13). Both AVR–Pia and AVR1–CO39 physically interact with RGA5–HMA, and this interaction is required for triggering resistance (13, 26).

Despite similarities in the Pik-1/Pik-2 and RGA5/RGA4 systems, their mechanisms of activation are different. The Pik-1/Pik-2 pair appears to use a cooperative mechanism, where effector recognition by the HMA in the sensor NLR Pik-1 requires the helper NLR Pik-2 to initiate signaling, but Pik-2 cannot signal on its own. Contrastingly, the RGA5/RGA4 pair functions via negative regulation, where recognition of the effector through RGA5–HMA derepresses signaling by RGA4 (27, 28). However, details of the NLR interactions and the resultant downstream signaling remain to be understood.

The interface between AVR–Pik effectors and the HMA domain of both Pikp and Pikm has been extensively studied and structurally characterized (11, 12). Recently, the structure of AVR1–CO39 in complex with the HMA domain of RGA5 was also elucidated (29), and it revealed that the HMA/effector interface was substantially different compared with the Pik NLR pairs. This has raised intriguing questions concerning how structurally similar but sequence-divergent HMA domains distinguish between structurally similar but sequence-divergent pathogen effectors.

Here, we reveal that Pikp is able to trigger partial disease resistance to the “mis-matched” effector AVR–Pia in rice and elicits a weak cell death response in *Nicotiana benthamiana*. Pikp–HMA binds AVR–Pia *in vitro*, at the RGA5/AVR1–CO39-like interface, rather than the Pik/AVR–Pik-like interface. This structural understanding of effector cross-reactivity in the Pik/RGA systems provides insights into the evolution and function of integrated HMA domains in NLRs. It also hints at the potential to engineer the HMA of Pikp to respond robustly to both AVR–PikD and AVR–Pia at the different interfaces.

## Results

### Rice plants expressing Pikp are partially resistant to M. oryzae expressing AVR–Pia

**Figure 1.**
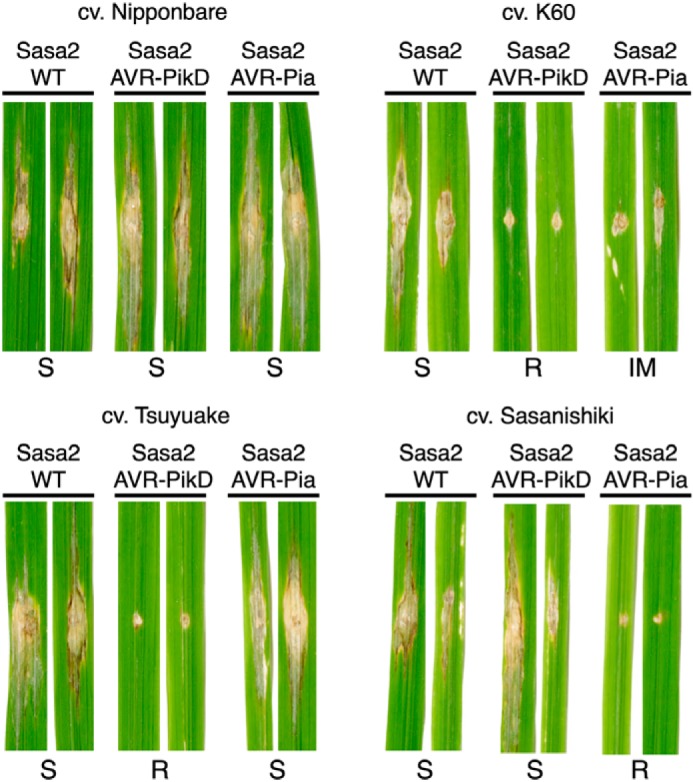
**Pikp confers partial resistance to *M. oryzae* expressing AVR–Pia.** Images of rice leaves following spot-inoculation assays of Sasa2 *M. oryzae* strain expressing no effectors (WT), AVR–PikD, or AVR–Pia. Strains were inoculated onto rice cultivars containing either Pikp-1/Pikp-2 (cv. K60), Pikm-1/Pikm-2 (cv. Tsuyuake), RGA5/RGA4 (cv. Sasanishiki) or none of the above (cv. Nipponbare). *S* = susceptible; *R* = resistant; *IM* = intermediate, and all are qualitative phenotype descriptors based on observations. Leaf samples were harvested 10 days post-inoculation. The assays were repeated at least three times with similar results.

We used a spot-inoculation assay to infect rice cultivars with a pathogen strain (Sasa2) transformed to express different effectors. As expected, rice plants that do not express either Pik or RGA NLRs (cv. Nipponbare) are susceptible to infection by all *M. oryzae* Sasa2 lines tested (clear spreading lesions away from the infection site, Fig. 1). Rice plants expressing Pikp (cv. K60) showed resistance to the Sasa2 lines expressing AVR–PikD (positive control) and consistently displayed a qualitatively reduced susceptibility (partial resistance) phenotype to lines expressing AVR–Pia, developing disease lesions that spread away from the infection site, but are not as developed as the negative controls. This partial resistance phenotype was not observed in rice plants expressing Pikm (cv. Tsuyuake), consistent with results from *N. benthamiana*. Furthermore, rice plants expressing RGA5/RGA4 (cv. Sasanishiki) are susceptible to the Sasa2 line expressing AVR–PikD, showing these NLRs do not partially respond to this effector. All pairwise resistance phenotypes behaved as expected.

### Co-expression of Pikp/AVR–Pia in N. benthamiana elicits a weak cell death response

**Figure 2.**
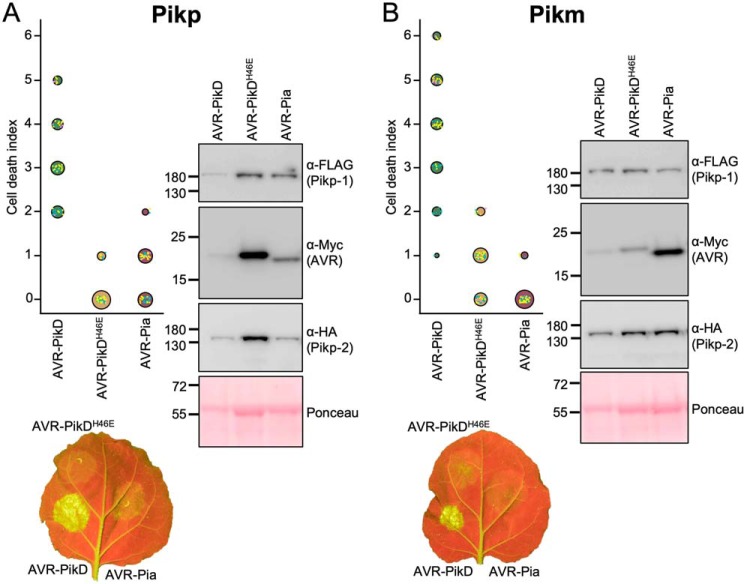
**Pikp, but not Pikm, responds weakly to AVR–Pia when transiently expressed in *N. benthamiana*.** *N. benthamiana* leaves were visually scored for macroscopic cell death 5 days post-infiltration using the previously published scoring scale (11) from 0 to 6. Representative leaf image shows cell death as autofluorescence under UV light (note: data not used for dot plot). Dot plots each show 70 repeats of the cell-death assay (10, 30, and 30 technical repeats over three independent experiments). The size of the center dot at each cell death value is directly proportional to the number of replicates in the sample with that score. All individual data points are represented as *dots*, colored by independent repeats. Western blottings show protein accumulation following transient expression in *N. benthamiana* 5 days post-agroinfiltration and are representative of three biological repeats (the amount of protein in the Pik-1/Pik-2/AVR–PikD samples appears lower (as indicated in the Ponceau image for total loading) due to greater cell death in this sample, limiting protein accumulation). *A,* Pikp-1/Pikp-2 transiently expressed with AVR–PikD, AVR–PikD^H46E^, and AVR–Pia. *B,* Pikm-1/Pikm-2 transiently expressed with AVR–PikD, AVR–PikD^H46E^, and AVR–Pia.

*N. benthamiana* is a well-established model system for assaying the response of rice NLRs to *M. oryzae* effectors (11, 12, 28). Therefore, we used this system to test whether Pik NLRs would show any response to the effector AVR–Pia. When AVR–Pia was transiently expressed in *N. benthamiana* via agroinfiltration, along with Pikp-1 and Pikp-2, there was a weak cell death response observed, as visualized by a yellowing of the tissue at the infiltration site, and fluorescence under UV light (Fig. 2*A*). The cell death was weaker compared with AVR–PikD (positive control), but it was stronger than for the AVR–PikD point mutant (AVR–PikD^H46E^), a negative control that is not recognized by Pikp (11). To confirm that each protein was expressed, Western blot analysis of extracted leaf tissue was used to assess protein accumulation (Fig. 2*A*). These results show that the Pikp NLRs can respond to AVR–Pia, although the response was limited compared with their “matched” effector AVR–PikD.

Interestingly, when the Pikm-1/Pikm-2 pair was tested against the same effectors (AVR–PikD, AVR–PikD^H46E^, and AVR–Pia), there was no macroscopic cell death observed to AVR–Pia *in planta*, despite confirmed expression of all proteins in the leaf tissue (Fig. 2*B*). There was a weak response to the AVR–PikD^H46E^ negative control, as observed previously, due to differences in the AVR–PikD His-46 interface with Pikm–HMA compared with Pikp–HMA (12). This suggests that the weak cell death response to AVR–Pia is specific for the Pikp allele.

### HMA domain of Pikp can bind AVR–Pia in vitro

Previously, a tight correlation was observed between *in planta* response phenotypes in *N. benthamiana* and rice, and *in vitro* binding between Pik–HMA domains and effectors (12, 18). We therefore tested the interaction of Pikp–HMA and Pikm–HMA domains with AVR–Pia following heterologous expression and purification of these proteins.

**Figure 3.**
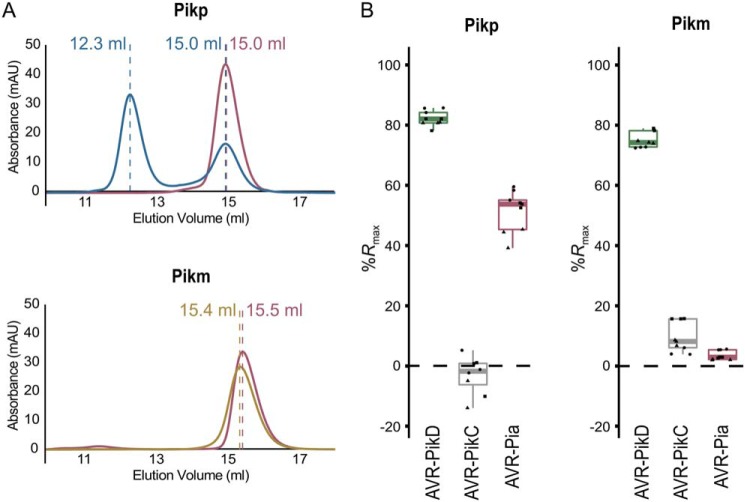
**Pikp–HMA, but not Pikm–HMA, binds AVR–Pia *in vitro*.** *A,* analytical gel-filtration traces assessing complex formation of Pikp–HMA (*top panel*) and Pikm–HMA (*bottom panel*) with AVR–Pia. Elution volumes for AVR–Pia alone (*pink*) and when mixed with Pikp–HMA (*blue*) and Pikm–HMA (*gold*) are labeled. Earlier elution indicates a larger molecular mass. The void volume of the column is 7.4 ml. SDS-PAGE analysis of eluent at the relevant volumes is shown in Fig. S1. The absorbance observed is only due to the effectors, as Pik–HMA domains do not absorb light at the wavelength measured. The interaction between Pik–HMAs and AVR–PikD was shown previously (11, 12). *B,* surface plasmon resonance data showing *R*_max_ (%) (the percentage of theoretical maximum response for HMA binding to immobilized effector) for Pikp–HMA (*left panel*) and Pikm–HMA (*right panel*) at 100 nm concentration binding to AVR–PikD, AVR–PikC, or AVR–Pia. Based on previously published data (12), binding was assumed to be 2:1 for Pikp–HMA with AVR–PikD and AVR–PikC, and 1:1 for all other interactions. Box plots show data for three repeats carried out in triplicate, where data points for each repeat are shown as a different shape. Note that only eight data points are shown for Pikp–HMA with the negative control AVR–PikC, due to poor effector capture in a single run. Equivalent data for 40 and 4 nm HMA concentrations are shown in Figs. S1 and S2.

First, analytical gel filtration was used to qualitatively determine whether Pik–HMA domains and AVR–Pia could form a complex. In isolation, AVR–Pia elutes at a retention volume of 15–15.5 ml (Fig. 3*A*). When mixed with the Pikm–HMA domain, no change in AVR–Pia retention was observed, consistent with the lack of response in plants. By contrast, when mixed with the Pikp–HMA domain, AVR–Pia elutes earlier at ∼12 ml suggesting a complex is formed, which was confirmed by SDS-PAGE (Fig. S1). Note that Pik–HMA domains do not sufficiently absorb UV light to give a signal in gel filtration under the conditions shown, but it can be seen by SDS-PAGE.

We then used surface plasmon resonance (SPR) to measure binding affinities, as described previously (12). These results were expressed as a percentage of the theoretical maximum response (*R*_max_), which gives a relative indication of binding strength. The positive and negative controls for Pikp–HMA and Pikm–HMA binding, the effector variants AVR–PikD and AVR–PikC, show strong and weak/no binding, as expected (Fig. 3*B* and Figs. S1 and S2). Consistent with gel filtration, essentially no binding is observed between Pikm–HMA and AVR–Pia, but Pikp–HMA binds AVR–Pia at ∼50% *R*_max_ (for the 100 nm Pikp–HMA concentration), independently confirming *in vitro* interaction and correlating with *in planta* responses.

### Pikp–HMA binds AVR–Pia at a different interface to AVR–PikD

To visualize the interface formed between Pikp–HMA and AVR–Pia, and compare it to that with AVR–Pik, we purified the complex between these proteins and determined the structure to 1.9 Å resolution using X-ray crystallography. The details of X-ray data collection, structure solution, and structure completion are given under “Experimental procedures,” Table 1, and Fig. S3.

**Table 1:** **X-ray data collection and refinement statistics for Pikp–HMA/AVR–Pia**

Data collection statistics	
Wavelength (Å)	0.9763
Space group	*P*22_1_2_1_
Cell dimensions	
*a*, *b*, *c* (Å)	34.84, 53.44, 117.81
α, β, γ (○)	90.00, 90.00, 90.00
Resolution (Å)*^a^*	48.67–1.90 (1.94–1.90)
*R*_merge_ (%)*^b^*	5.7 (122.9)
Mean *I*/σ*I^b^*	19.7 (2.4)
Completeness (%)*^b^*	100 (100)
Unique reflections*^b^*	18,107 (1151)
Redundancy*^b^*	12.6 (13.3)
*CC*(1/2) (%)*^b^*	99.9 (80.9)
**Refinement and model statistics**	
Resolution (Å)	48.72–1.90 (1.95–1.90)
*R*_work/_*R*_free_ (%)*^c^*	20.3/24.5 (35.8/41.8)
No. of atoms	
Protein	2113
Water	89
Average *B*-factors (Å^2^)	
Protein	54.1
Water	58.1
R.m.s deviations*^c^*	
Bond lengths (Å)	0.0117
Bond angles (°)	1.501
Ramachandran plot (%)*^d^*	
Favored	98.5
Allowed	1.5
Outliers	0
MolProbity score	1.52 (95th percentile)

*^a^* The highest resolution shell is shown in parentheses.

*^b^* Date were calculated by Aimless.

*^c^* R.m.s. is root mean square. Data were calculated by Refmac5.

*^d^* Data were calculated by MolProbity.

**Figure 4.**
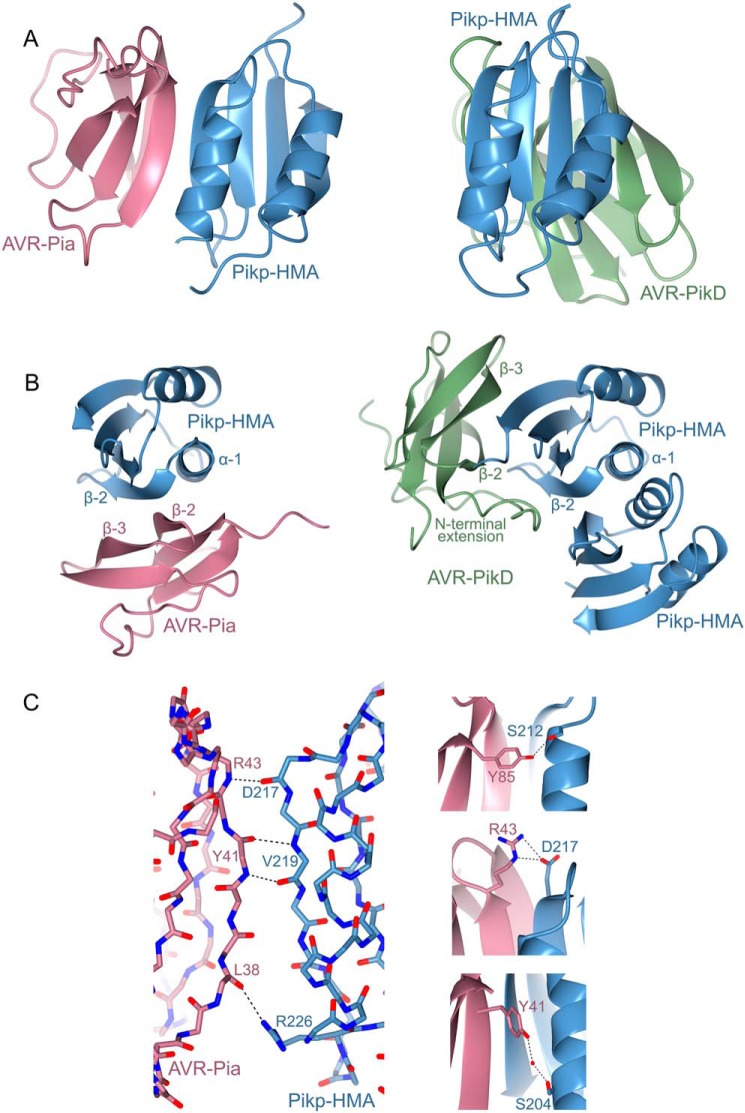
**Structural basis of Pikp–HMA interaction with AVR–Pia.** *A,* schematic diagram of the structure of Pikp–HMA in complex with AVR–Pia refined to 1.9 Å resolution by X-ray crystallography (*left*), compared with the structure of Pikp–HMA in complex with AVR–PikD (PDB code 6G10, *right*, only a Pikp–HMA monomer is displayed here). AVR–Pia is shown in *pink*, AVR–PikD in *green,* and Pikp–HMA in *blue*. The Pikp–HMA monomer is shown in the same orientation for both structures. *B,* alternative view (rotated ∼90 °C horizontally and vertically) of the Pikp–HMA/AVR–Pia and Pikp–HMA/AVR–PikD structures shown in *A*, with secondary structure features labeled (Pikp–HMA dimer structure shown in this view). *C,* details of the interface between Pikp–HMA and AVR–Pia, showing interactions at the peptide backbone (*left*), and selected side-chain interactions (*right*). *Dotted lines* show hydrogen bonds, and *red spheres* represent water molecules. Carbons are colored according to the protein (Pikp–HMA in *blue* and AVR–Pia in *pink*) with oxygen atoms shown in *red* and nitrogen in *dark blue. Labels* show the single letter amino acid code with position in the peptide chain. Bond distances for hydrogen bonds shown are 2.80, 3.05, 2.81, and 3.06 Å (*left panel, top to bottom*), and 2.87 Å (*right panel, top*), 3.0/2.86 Å (*right panel, middle*), and 2.66/3.05 Å (*right panel, bottom*).

Each partner in the complex adopts a similar overall fold to previously solved structures. Pikp–HMA (11, 12) comprises two adjacent α-helices opposite a four-stranded β-sheet (Fig. 4, *A* and *B*). Previous structures of AVR–Pia were determined by NMR spectroscopy (18, 30), and the crystal structure determined here is very similar (0.92 Å over 65 aligned residues), comprising the six-stranded β-sandwich characteristic of MAX effectors (18). In the crystal structure, β-5 is not well-defined and appears as a loop joining β-4 and β-6, but overall the configuration of this region is similar to the NMR ensemble. As observed previously, a disulfide bond is formed between residues Cys-25 and Cys-66.

Strikingly, although the two proteins in the complex adopt essentially identical folds to their structures in isolation, Pikp–HMA binds AVR–Pia at a completely different interface to the AVR–Pik effectors (Fig. 4, *A* and *B*). Whereas Pikp–HMA binds AVR–PikD opposite the face of its β-sheet, it binds AVR–Pia adjacent to α-1 and β-2 (Fig. 4*B*). In both cases, the position of Pikp–HMA relative to the effector allows the formation of a continuous anti-parallel β-sheet between the proteins (Fig. S4). In the case of AVR–PikD, the β-strands from Pikp–HMA form a sheet with β-strands 3–5 of AVR–PikD. For AVR–Pia, the β-strands involved are 1, 2, and 6. Another striking feature is that whereas Pikp–HMA is a dimer in the structure with AVR–PikD (11, 12), it is a monomer with AVR–Pia. Indeed, AVR–Pia occupies the same binding surface as the Pikp–HMA dimer in the Pikp–HMA/AVR–PikD structure, which suggests that AVR–Pia binding is competing with Pikp–HMA dimerization in solution.

The interface formed between Pikp–HMA and AVR–Pia covers an area of 460 Å^2^ (as calculated by PISA (31)), approximately half of that seen between Pikp–HMA and AVR–PikD (986 Å^2^ (12)). Furthermore, the interface between Pikp–HMA and AVR–Pia is dominated by hydrogen bonds between the peptide backbone, with the main contributions derived from Pikp–HMA^Asp-217^, Pikp–HMA^Val-219^, AVR–Pia^Tyr-41^, and AVR–Pia^Arg-43^ (Fig. 4*C*). The backbone oxygen atom of AVR–Pia^Leu-38^ also forms a hydrogen bond with the side chain of Pikp–HMA^Arg-226^. There are only limited side-chain–mediated interactions in the Pikp–HMA/AVR–Pia complex, with a hydrogen bond/salt bridge interaction formed between AVR–Pia^Arg-43^ and Pikp^Asp-217^, and the hydroxyl group on the C-terminal residue of AVR–Pia, Tyr-85, also forms a hydrogen bond with Pikp^Ser-212^ (Fig. 4*C*). Finally, an indirect interaction, mediated by a water molecule, is found between the side chains of AVR–Pia^Tyr-41^ and Pikp^Ser-204^ (Fig. 4*C*). These limited intermolecular interactions and small interface area provide an explanation for the weaker binding affinity seen for Pikp–HMA to AVR–Pia when compared with AVR–PikD *in vitro* (Fig. 3*B* and Figs. S1 and S2) and reduced responses *in planta*.

### Pikp recognizes AVR–Pia through different molecular features compared with AVR–PikD

Despite only sharing 17% sequence identity (Fig. S5), AVR–Pia and AVR–PikD both adopt the MAX effector fold. However, AVR–PikD also contains an additional N-terminal extension (comprising residues Arg-31 to Pro-52) that partially wraps around and is held in place by the core structure (see Fig. 4*B* and Fig. S5). This extension plays a key role in the interaction of AVR–PikD and Pikp–HMA, including a histidine residue (His-46), which forms hydrogen bond/salt bridge interactions with Ser-218 and Glu-230 in Pikp–HMA (11). We considered that modifying the core MAX fold of AVR–Pia, to add the AVR–PikD N-terminal extension, might allow Pikp to respond more strongly to the effector by switching the interaction of the chimeric effector (AVR–Pia^NAVR–PikD^) to the “AVR–PikD-like” interface of Pikp–HMA. We also investigated the effect of removing the N-terminal extension from AVR–PikD (AVR–PikD^Δ22–52^).

**Figure 5.**
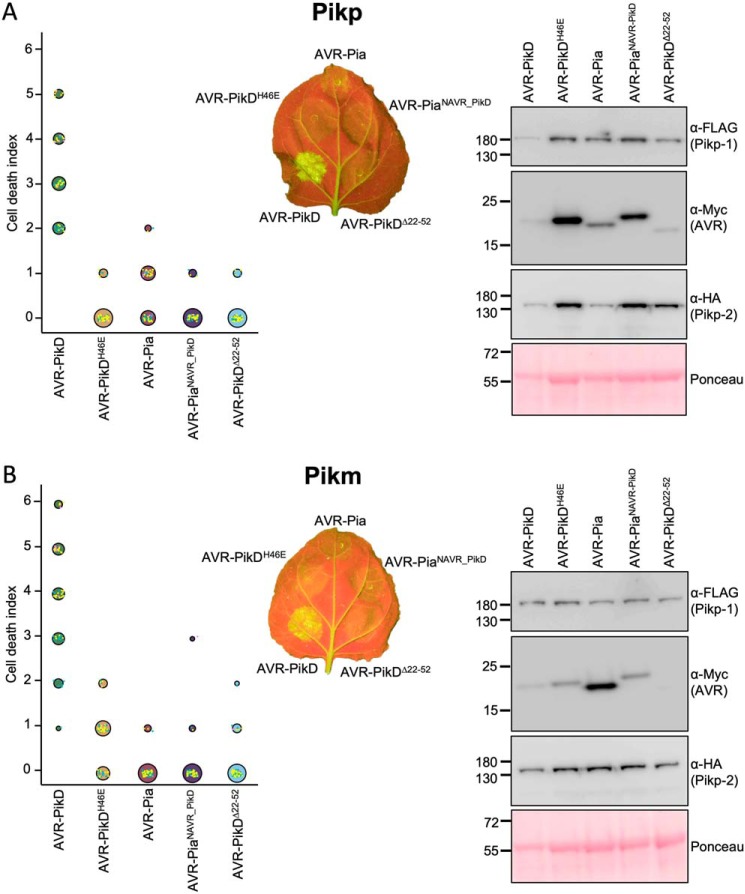
**Modifying AVR–Pia with the N-terminal extension of AVR–PikD does not affect the Pik NLR response.** *N. benthamiana* leaves were visually scored for cell death 5 days post-infiltration using the previously published scoring scale (11) from 0 to 6. Representative leaf image shows cell death as autofluorescence under UV light. Dot plots each show 70 repeats of the cell-death assay (10, 30, and 30 technical repeats over three independent experiments). The size of the center dot at each cell death value is directly proportional to the number of replicates in the sample with that score. All individual data points are represented as dots, colored by independent repeat. Western blots show protein accumulation following transient expression in *N. benthamiana* 5 days post-agroinfiltration and are representative of three biological repeats (the amount of protein in the Pik-1/Pik-2/AVR–PikD samples appears lower (as indicated in the Ponceau image for total loading) due to greater cell death in this sample, limiting protein accumulation). *A,* Pikp-1/Pikp-2 transiently expressed with AVR–PikD, AVR–PikD^H46E^, AVR–Pia, AVR–Pia^NAVR–PikD^, and AVR–PikD^Δ22–52^. *B,* Pikm-1/Pikm-2 transiently expressed with AVR–PikD, AVR–PikD^H46E^, AVR–Pia, AVR–Pia^NAVR–PikD^, and AVR–PikD^Δ22–52^. The data shown for AVR–PikD, AVR–PikD^H46E^, and AVR–Pia is the same as shown in Fig. 2, to give direct comparison (all of these data were acquired within the same experimental repeats).

After generating the appropriate constructs, they were expressed in *N. benthamiana* via agroinfiltration alongside Pikp-1/Pikp-2 or Pikm-1/Pikm-2. In these assays, neither Pikp nor Pikm responded to either AVR–Pia^NAVR–PikD^ or AVR–PikD^Δ22–52^ (Fig. 5). Western blot analysis showed that accumulation of AVR–PikD^Δ22–52^ in the leaf tissue is low, suggesting that the N-terminal truncation has destabilized AVR–PikD (Fig. 5). However, we confirmed the expression of AVR–Pia^NAVR–PikD^ in the infiltrated leaf tissue, suggesting that the lack of cell death in this case is not due to lack of protein accumulation (Fig. 5). It is possible that AVR–Pia^NAVR–PikD^ retains interaction at the “AVR–Pia-like” interface, but the presence of a disordered N-terminal extension hinders response in the full-length protein (the N terminus cannot adopt the same conformation as AVR–PikD at the AVR–Pia-like interface as this would generate a steric clash, see Fig. S6).

## Discussion

Integrated domains in plant NLR immune receptors bait pathogen effectors to initiate an immune response. Understanding the specificity of effector binding by these integrated domains gives important insights into evolution and function of plant innate immunity. The discovery that rice blast pathogen effectors with a common structural fold can be recognized by the same type of integrated domain in rice NLRs raises questions about specificity, and possible plasticity, of recognition. *M. oryzae* MAX effectors AVR–PikD and AVR1–CO39 are bound at different interfaces by their respective NLR-encoded HMA domains (11, 12, 29). Here, we investigated the interaction of a mis-matched NLR integrated domain (Pikp–HMA) and a pathogen effector (AVR–Pia) to better understand how protein interfaces contribute to signaling. Ultimately, we hope such studies will lead to improved engineering of NLRs for use in crops.

### Single NLR integrated domain can bait distinct pathogen effectors

**Figure 6.**
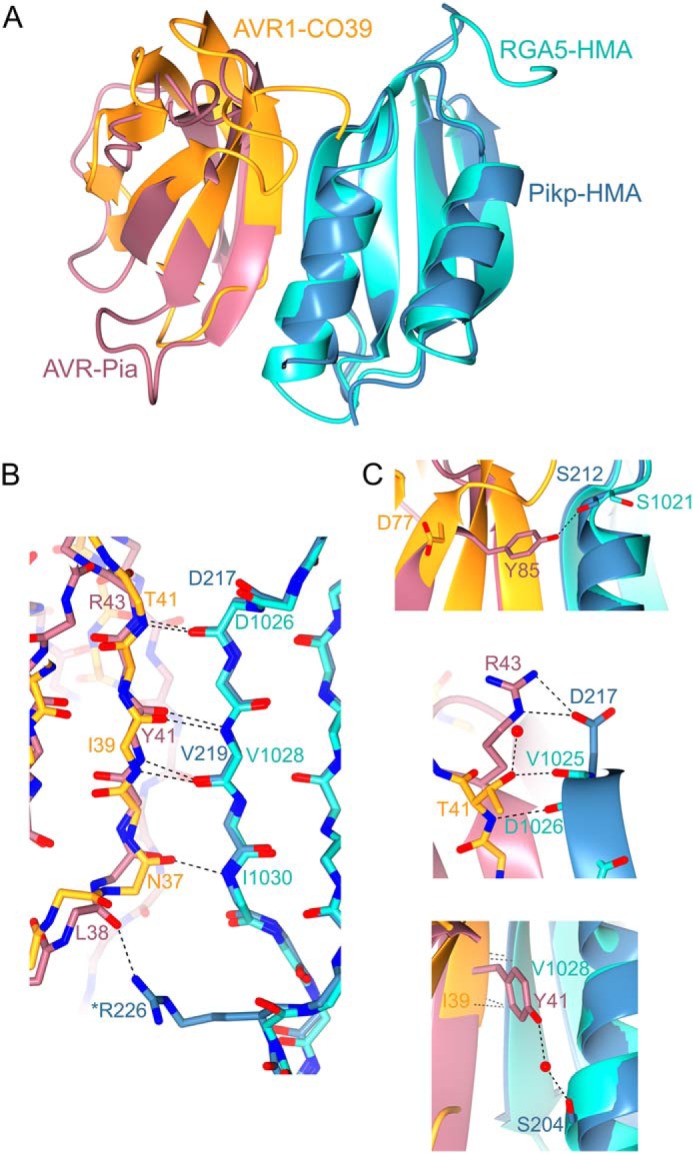
**Structural comparison of Pikp–HMA/AVR–Pia and RGA5–HMA/AVR1–CO39 complexes.** Overlays of Pikp–HMA/AVR–Pia with RGA5–HMA/AVR1–CO39 (PDB code 5ZNG) are superposed on the HMA domain (root mean square deviation 0.81 Å over 73 residues). AVR–Pia is shown in *pink*, Pikp–HMA in *blue*, AVR1–CO39 in *orange,* and RGA5–HMA in *turquoise. A,* cartoon ribbon structure represents overall structures. *B,* details of interactions between the peptide backbones at the interface. *Dotted lines* show hydrogen bonds, and carbons are colored according to the chain with oxygen atoms shown in *red* and nitrogen in *dark blue. Labels* show the single letter amino acid code (colored according to protein) with position in the peptide chain. * indicates a side chain, rather than backbone interaction. *C,* further details of important interactions are at the interfaces. *Red spheres* represent water molecules.

Intriguingly, although Pikp–HMA binds AVR–Pia at a different interface to AVR–PikD, it uses the same interface that RGA5–HMA uses to bind AVR1–CO39 (29). Therefore, a single integrated domain in a plant NLR can interact with divergent effectors via different surfaces. Fig. 6 shows a comparison between the Pikp–HMA/AVR–Pia complex and that of the published RGA5–HMA/AVR1–CO39 structure (29) (HMA sequence alignments shown in Fig. S5). Like Pikp–HMA, RGA5–HMA forms a dimer in solution, and binding to the effector competes with this, such that only an HMA monomer is present in each complex (29). Globally, the complexes are very similar, and both rely heavily on peptide backbone interactions for maintaining an interaction between the HMA and effector. One of the most striking differences is the contribution of residues in the N terminus of AVR1–CO39 (Trp-23 and Lys-24) to the interaction, which is not seen in the Pikp–HMA/AVR–Pia complex. However, the three important binding regions in the RGA5–HMA/AVR1–CO39 complex noted by Guo *et al.* (29) are shared by Pikp–HMA/AVR–Pia, although the nature of the residues and interactions involved differ. At the equivalent AVR1–CO39^Thr-41^ and RGA5^Asp-1026^ binding area, there is a side-chain interaction between AVR–Pia^Arg-43^ and Pikp^Asp-217^. At an equivalent location to the second binding area (AVR1–CO39^Ile-39^ and RGA5^Val-1028^), there are AVR–Pia^Tyr-41^ and Pikp^Val-219^ backbone interactions and a water-mediated hydrogen bond between the side chain of AVR–Pia^Tyr-41^ and Pikp^Ser-204^. Finally, the third binding area involves a backbone interaction between RGA5–HMA^Ile-1030^ and AVR1–CO39^Asn-37^. At a similar area in the Pikp–HMA/AVR–Pia interface, there is a hydrogen bond between the backbone of AVR–Pia^Leu-38^ and the side chain of Pikp^Arg-226^. The overall close similarities between these complexes implies that this is a biologically relevant interface and supports binding studies that AVR–Pia also interacts with RGA5–HMA at this interface (29).

Whereas the different HMA domains of RGA5 and Pik use different interfaces to interact with their cognate effectors, Pikp has the capacity to use both of these for binding different effectors. Our initial observations in rice suggest that RGA5/RGA4 cannot respond to AVR–PikD, indicating that RGA5 might not be able to use the alternative “AVR–PikD-like” binding interface. We hypothesize that following HMA domain integration into their respective ancestor proteins, Pik-1 and RGA5 have evolved to respond to their cognate effectors through variation both within the HMA domains but also within the rest of the NLR architecture. The position of the HMA domain integration is likely critical and may affect available HMA-binding interfaces for both the effectors and intra-/inter-molecular interactions within the NLRs that support downstream signaling.

### Pik NLR response to and interaction with AVR–Pia is allele-specific

Pikm is not able to respond to AVR–Pia, despite both Pikp and Pikm recognizing the same MAX effector AVR–PikD. When the structure of the Pikp–HMA/AVR–Pia complex is overlaid with Pikm–HMA (12), the overall HMA conformation is virtually identical, but sequence diversity results in different side chains being presented at the predicted interaction surface. Most apparent is that Pikp–HMA^Asp-217^, which forms a hydrogen bond/salt bridge interaction with AVR–Pia^Arg-43^ (Figs. 4*C* and 6*C* and Fig. S3), is replaced by a histidine residue at the equivalent position in Pikm–HMA. This change may, in part, account for a reduced affinity for AVR–Pia, although it seems unlikely to fully account for a lack of interaction. Further experiments are required to investigate why Pikm–HMA does not bind AVR–Pia *in vitro* or Pikm respond to AVR–Pia in planta.

### Using integrated domain cross-reactivity for NLR engineering

The cross-reactivity of Pikp for the mis-matched AVR–Pia effector raises exciting possibilities around engineering Pikp to respond more robustly to this effector, while maintaining AVR–PikD interactions. As noted by Guo *et al.* (29), the use of different interfaces for the effectors may allow engineering of one surface without significantly disrupting the binding at the other. Such detailed structural knowledge paves the way toward future NLR engineering for improved disease resistance that may be applicable to other NLR/effector pairs.

## Experimental procedures

### Cloning and construct generation

Constructs for *N. benthamiana* cell-death assays were generated by Golden Gate cloning methods (32). Domesticated Pik-1 and Pik-2 NLRs were used as described in de la Concepcion (12), and each effector construct was generated with an N-terminal 4× Myc tag, a Ubi10 promoter (from *Arabidopsis thaliana*), and 35S terminator.

For *in vitro* studies, isolated Pikp–HMA (residues 186–263) and Pikm–HMA (residues 186–264) domain constructs were used as described in de la Concepcion *et al.* (12). For analytical gel-filtration and crystallography studies, AVR–Pia (residues 20–85) was cloned into the pOPINS3C vector by In-Fusion cloning (33) to yield a cleavable N-terminal His_6_-SUMO–tagged construct. For surface plasmon resonance, effectors were amplified from pOPINS3C and cloned into pOPINE to yield a noncleavable C-terminal His_6_-tag in addition to the SUMO tag, following the strategy used in Ref. 11.

### N. benthamiana cell-death assays

Transient *in planta* expression, cell-death assays, and confirmation of protein expression was carried out as described by de la Concepcion *et al.* (12). Briefly, *Agrobacterium tumefaciens* GV3101 was used to deliver T-DNA constructs into 4-week-old *N. benthamiana* plants (grown at high-light intensity, 22–25 °C). Pik-1, Pik-2, AVR–Pik, and the P19 suppressor of silencing were mixed prior to infiltration and delivered at *A*_600_ 0.4, 0.4, 0.6, and 0.1 respectively. At 5 dpi, detached leaves were imaged under UV light on the abaxial side and visually scored against a cell-death index described previously (11). Scores from three independent repeats (comprising 10, 30, and 30 internal repeats) are shown as dot plots, generated using R (34) and graphics package ggplot2 (35). The size of the center dot at each cell death value is directly proportional to the number of replicates in the sample with that score. All individual data points are represented as dots, colored by independent repeat.

To confirm expression of relevant proteins, leaf disks taken from representative infiltration spots were frozen, ground, and mixed with 2× w/v extraction buffer (25 mm Tris, pH 7.5, 150 mm NaCl, 1 mm EDTA, 10% v/v glycerol, 10 mm DTT, 2% w/v polyvinylpolypyrrolidone, 0.1% Tween® 20, 1× plant protease inhibitor mixture (Sigma)). These samples were then centrifuged (20,000 × *g* at 4 °C for 5 min), and the supernatant was decanted and centrifuged again for a further 2 min. 20 μl of sample was mixed with 8 μl of SDS-PAGE loading dye. Following SDS-PAGE, protein samples were transferred to polyvinylidene difluoride membrane using a trans-blotter. Membranes were blocked with TBS-T (50 mm Tris-HCl, pH 8.0, 150 mm NaCl, 0.1% Tween 20) supplemented with 5% w/v dried milk powder for at least 60 min at 4 °C. Blots were then probed with relevant antibody conjugates to epitope tags, α-FLAG–HRP (Generon, 1:5000 dilution used), α-Myc–HRP (Santa Cruz Biotechnology, 1:1000 dilution used), or α-HA–HRP (Thermo Fisher Scientific, 1:3000 dilution used), washed, and developed with LumiBlue ECL Extreme reagents (Expedeon). Chemiluminescence was recorded using an ImageQuant LAS 500 spectrophotometer (GE Healthcare). Finally, blots were incubated with Ponceau stain to control for protein loading.

### Rice pathogenicity assays

*M. oryzae* strains Sasa2 and Sasa2 expressing *AVR–PikD* (the transformant harboring 22p:pex31-D (*AVR–PikD* allele fused with the promoter region of *AVR–Pia*)) used in this study are stored at the Iwate Biotechnology Research Center (21). To obtain protoplasts, hyphae of the Sasa2 strain were incubated for 3 days in 200 ml of YG medium (0.5% yeast extract and 2% glucose, w/v). Protoplast preparation and transformation with pex22p:pex22 (*AVR–Pia* fused with the promoter region of *AVR–Pia*) were performed as described previously (36) to generate Sasa2 strain expressing *AVR–Pia*. Bialaphos-resistant transformants were selected on plates with 250 μg/ml of Bialaphos (Wako Pure Chemicals).

Rice leaf blade spot inoculations were performed with *M. oryzae* strains as described previously (37). Disease lesions were scanned 14 dpi. The assays were repeated at least three times with qualitatively similar results.

### Expression and purification of proteins for in vitro studies

All proteins for *in vitro* studies were expressed from *E. coli* SHuffle cells (38) in auto-induction media (39). Cell cultures were grown at 30 °C for 5 h, followed by 16 °C overnight. Proteins were purified as described in Maqbool *et al.* (11).

Briefly, cells were 'harvested by centrifugation and resuspended in 50 mm Tris-HCl, pH 8.0, 500 mm NaCl, 50 mm glycine, 5% (v/v) glycerol, 20 mm imidazole supplemented with EDTA-free protease inhibitor tablets (Roche Applied Science). Cells were sonicated and, following centrifugation at 36,250 × *g* for 30 min, the clarified lysate was applied to a Ni^2+^-NTA column connected to an AKTA Xpress purification system (GE Healthcare). Proteins were step-eluted with elution buffer (50 mm Tris-HCl, pH 8.0, 500 mm NaCl, 50 mm glycine, 5% (v/v) glycerol, 500 mm imidazole) and directly injected onto a Superdex 75 26/60 gel-filtration column pre-equilibrated 20 mm HEPES, pH 7.5, 150 mm NaCl. Purification tags were removed by overnight incubation with 3C protease (10 μg/mg fusion protein) followed by passing through Ni^2+^-NTA (and for HMA domains MBP Trap HP columns (GE Healthcare)). The flow-through was concentrated as appropriate and loaded onto a Superdex 75 26/60 gel-filtration column for final purification and buffer-exchanged into 20 mm HEPES, pH 7.5, 150 mm NaCl. Purified protein was concentrated by ultrafiltration and stored at −80 °C.

### Expression and purification of proteins for crystallization

To prepare the Pikp–HMA/AVR–Pia complex for crystallization studies, separate cell cultures of SUMO-tagged AVR–Pia and His_6_–MBP-tagged Pikp–HMA were grown and harvested as described above. After initial protein purification and immediately following removal of the solubility tags, both proteins were combined and subsequently treated as a single sample for the final gel-filtration purification stage.

### Protein–protein interaction studies in vitro

Analytical gel filtration and surface plasmon resonance experiments were carried out as described by de la Concepcion *et al.* (12). For analytical gel filtration, purified proteins were run down a Superdex^TM^ 75 10/300 column (GE Healthcare) at 0.5 ml/min either alone or mixed to assess complex formation (mixtures were incubated on ice for 2 h prior to experiment). Effectors were used at 50 μm final concentration, and Pikp–HMA and Pikm–HMA were used at 100 and 50 μm, respectively, to account for dimer formation in solution. For surface plasmon resonance experiments, all proteins were prepared in SPR running buffer (20 mm HEPES, pH 7.5, 860 mm NaCl, 0.1% Tween 20). The C-terminal His_6_-tagged effector proteins were immobilized onto an NTA sensor chip (GE Healthcare) loaded into a Biacore T200 system (GE Healthcare) activated with 30 μl of 0.5 mm NiCl_2_ and giving a response of 250 ± 30. HMA protein was flowed over the immobilized effector at 30 μl/min (360 s contact time and 180 s dissociation time) at 4, 40, and 100 nm concentrations, considering HMA dimer formation where appropriate. The response of a reference cell was subtracted for each measurement. Raw data were exported; % *R*_max_ values were calculated in Microsoft Excel, and then individual % *R*_max_ data from three separate experiments were displayed as box plots in R. The sensor chip was regenerated between each cycle with an injection of 30 μl of 350 mm EDTA.

### Crystallization, data collection, and structure determination

For crystallization, the Pikp–HMA/AVR–Pia complex (in a buffer of 20 mm HEPES, 150 mm NaCl, pH 7.5) was used in sitting drop vapor-diffusion experiments. Drops were set up in 96-well plates, composed of 0.3 μl of purified protein (between 10 and 20 mg/ml) with 0.3 μl of reservoir solution, dispensed using the Oryx Nano crystallization robot (Douglas Instruments). Crystals for data collection were obtained in the Morpheus® screen (Molecular Dimensions), using protein at 18 mg/ml (measured by Direct Detect® spectrometer (Merck)). The crystals were found in well D2 of the screen, and the conditions in this well were as follows: 0.12 m alcohols (0.2 m 1,6-hexanediol; 0.2 m 1-butanol; 0.2 m 1,2-propanediol; 0.2 m 2-propanol; 0.2 m 1,4-butanediol; 0.2 m 1,3-propanediol), 0.1 m Buffer System 1 (1.0 m imidazole; MES monohydrate (acid), pH 6.5), and 50% v/v Precipitant Mix 2 (40% v/v ethylene glycol; 20% w/v PEG 8000). Crystals were frozen in liquid nitrogen, and X-ray data were collected at the Diamond Light Source (Oxfordshire) on beamline DLS-i03. Crystallographic data were processed using the Xia2 pipeline (40) and AIMLESS (41), as implemented in the CCP4 software suite (42). To solve the structure, a single model from the ensemble of AVR–Pia (PDB code 2MYW) and a monomer structure of Pikp–HMA (PDB code 5A6P) were used for molecular replacement in PHASER (43). COOT (44) was used for manual rebuilding, and successive rounds of manual rebuilding were followed by rounds of refinement using REFMAC5 (45). The structure was validated using the tools provided in COOT and finally assessed by MolProbity (46). All structure figures were prepared using the CCP4 molecular graphics program (CCP4MG) (42).

## Author contributions

F. A. V. and M. J. B. conceptualization; F. A. V., H. S., and K. Y. data curation; F. A. V., H. S., K. Y., and M. J. B. formal analysis; F. A. V., H. S., and M. J. B. validation; F. A. V., H. S., and K. Y. investigation; F. A. V. and H. S. visualization; F. A. V., H. S., K. Y., and M. F. methodology; F. A. V. and M. J. B. writing-original draft; F. A. V., H. S., S. K., R. T., and M. J. B. project administration; F. A. V., H. S., K. Y., M. F., S. K., R. T., and M. J. B. writing-review and editing; M. J. B. supervision; M. J. B. funding acquisition.

## Supplementary Material

Supporting Information

## Footnotes

2 The abbreviations used are:

NLR: nucleotide-binding leucine-rich repeat
HMA: heavy metal-associated
MBP: maltose-binding protein
HRP: horseradish peroxidase
SPR: surface plasmon resonance
PDB: Protein Data Bank
dpi: day post-inoculation
Ni^2+^-NTA: Ni^2+^-nitrilotriacetic acid.
