# Supplementary material for: Cross-reactivity of a rice NLR immune receptor to distinct effectors from the rice blast pathogen Magnaporthe oryzae provides partial disease resistance
Source: J Biol Chem. 2019 Jul 11;294(35):13006–16. doi: 10.1074/jbc.RA119.007730 (PMC6721932; doi:10.1074/jbc.RA119.007730)
Supplement: Supporting Information [file supp_294_35_13006__index.html]

Cross-reactivity of a rice NLR immune receptor to distinct effectors from the rice blast pathogen Magnaporthe oryzae provides partial disease resistance — Cross-reactivity of a rice NLR to blast effectors — Cross-reactivity of a rice NLR immune receptor to distinct effectors from the rice blast pathogen Magnaporthe oryzae provides partial disease resistance — Cross-reactivity of a rice NLR to blast effectors — Supporting Information 

# Cross-reactivity of a rice NLR immune receptor to distinct effectors from the rice blast pathogen *Magnaporthe oryzae* provides partial disease resistance

## Supporting Information

- Supporting Information (to be published online) - SI for publication
