# Supplementary material for: Cross-reactivity of a rice NLR immune receptor to distinct effectors from the rice blast pathogen Magnaporthe oryzae provides partial disease resistance
Source: J Biol Chem. 2019 Jul 11;294(35):13006–16. doi: 10.1074/jbc.RA119.007730 (PMC6721932; doi:10.1074/jbc.RA119.007730)
Supplement: Supporting Information [file supp_RA119.007730_143364_2_supp_358269_pl4832.pdf]

Cross-reactivity of a rice NLR immune receptor to distinct effectors from the blast pathogen leads to partial disease resistance

Freya A. Varden, Hiromasa Saitoh, Kae Yoshino, Marina Franceschetti, Sophien Kamoun, Ryohei Terauchi, Mark J. Banfield

Supporting Information

Figures S1 - S6

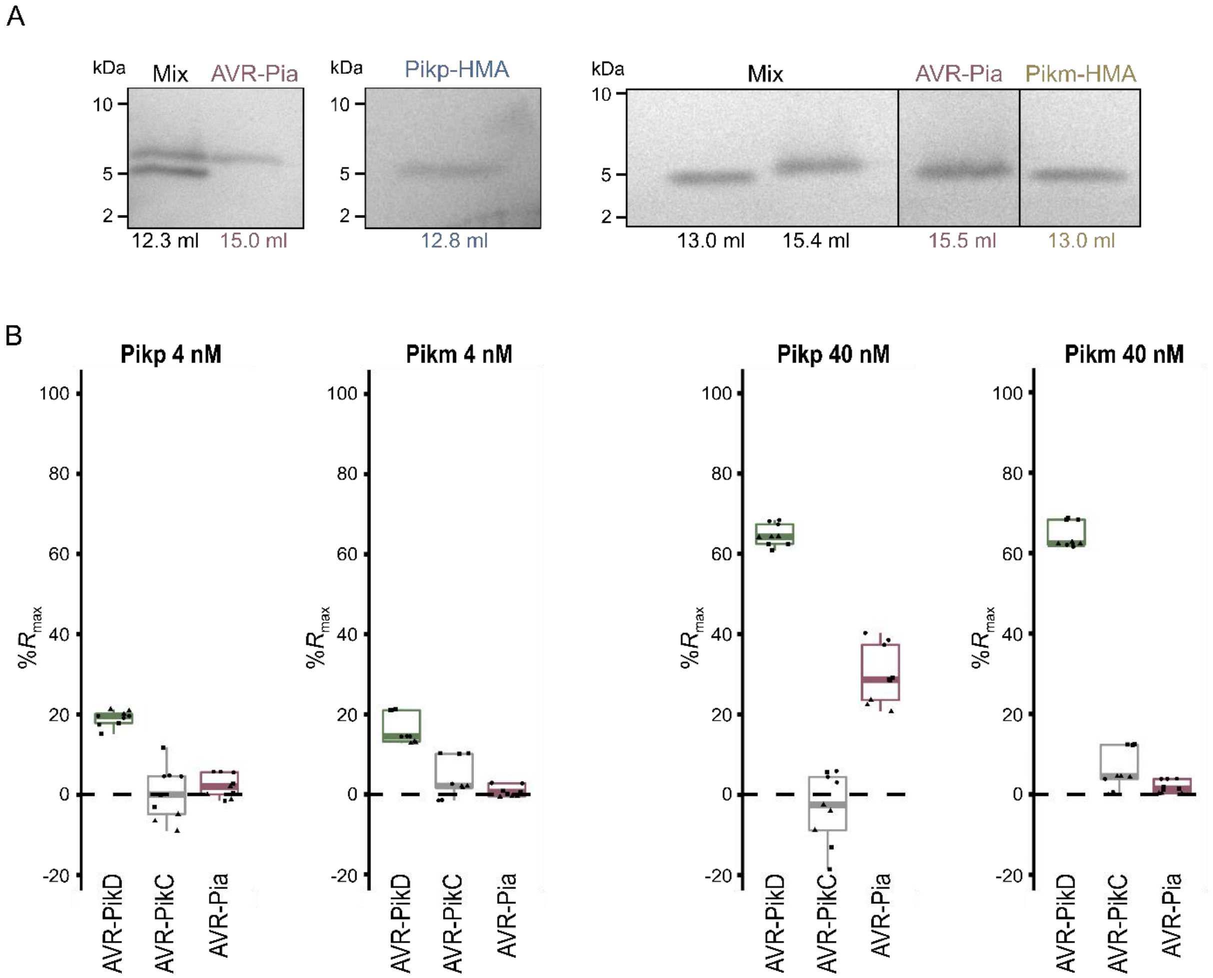

Figure S1. A) SDS-PAGE analysis showing the proteins eluted at the volumes indicated in the analytical gel filtration traces shown in Fig. 3A. The isolated HMA domains are not shown in Fig. 3A because they do not absorb sufficient UV light to be detected. B) Surface plasmon resonance R_{max} (%) data for Pikp-HMA and Pikm-HMA at 4 nM and 40 nM concentrations binding to AVR-PikD, AVR-PikC and AVR-Pia. Data is displayed in the same manner as shown in Fig. 3B.

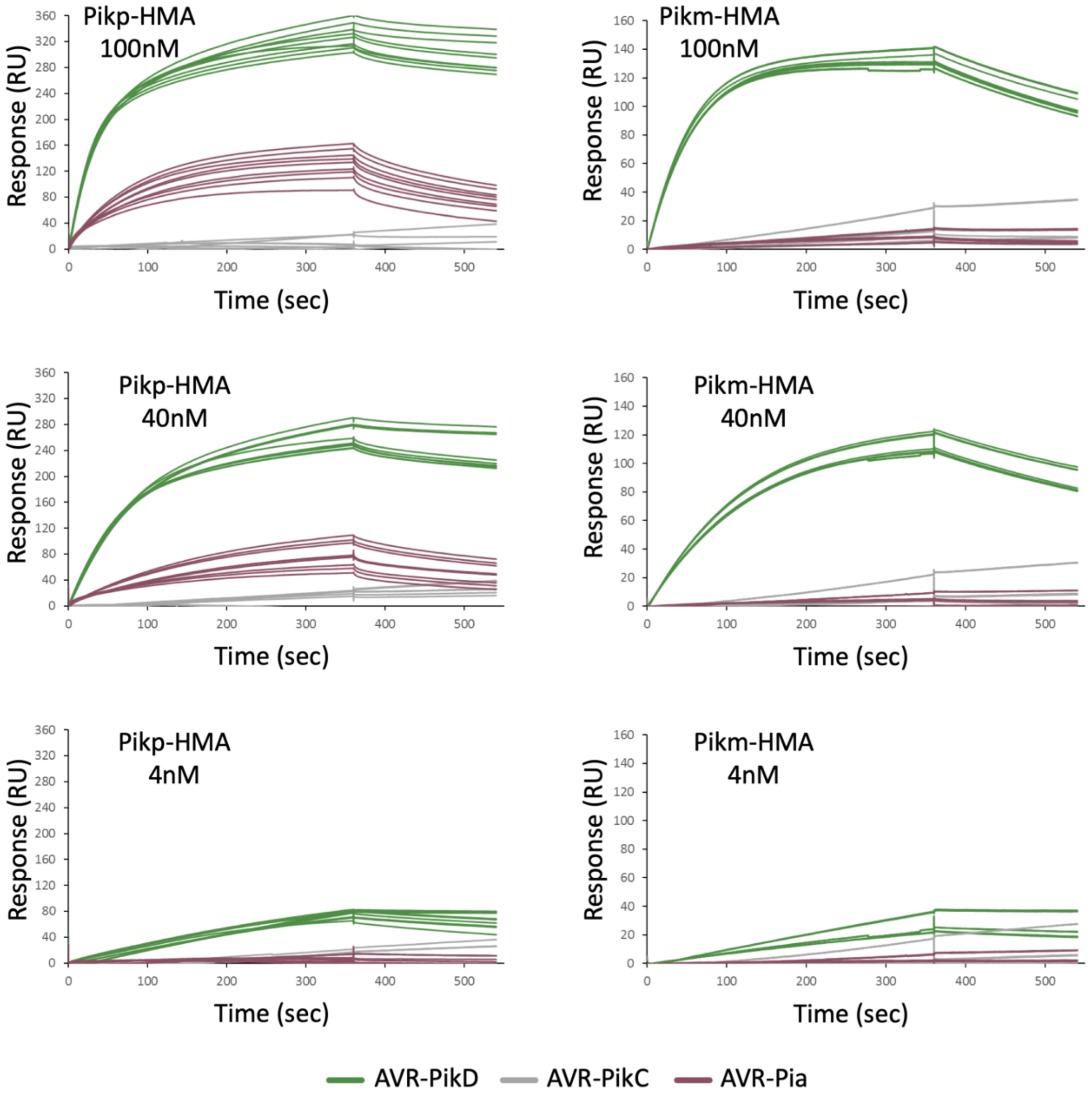

Figure S2. SPR traces for the interaction of Pikp-HMA (left) and Pikm-HMA (right) with AVR-PikD, AVR-PikC and AVR-Pia (as coloured). Three different concentrations of the Pik-HMA domains used are as shown (100 nM, top; 40 nM, middle; 4 nM, bottom).

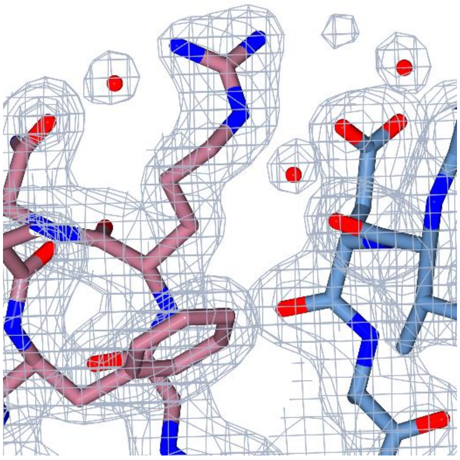

Figure S3. Image showing the Pikp-HMA/AVR-Pia structure modelled into electron density (shown in grey mesh), at an interface region between the two proteins (centred around AVR-Pia^{R43} and Pikp-HMA^{D217}). Atoms are coloured as described in Fig. 4.

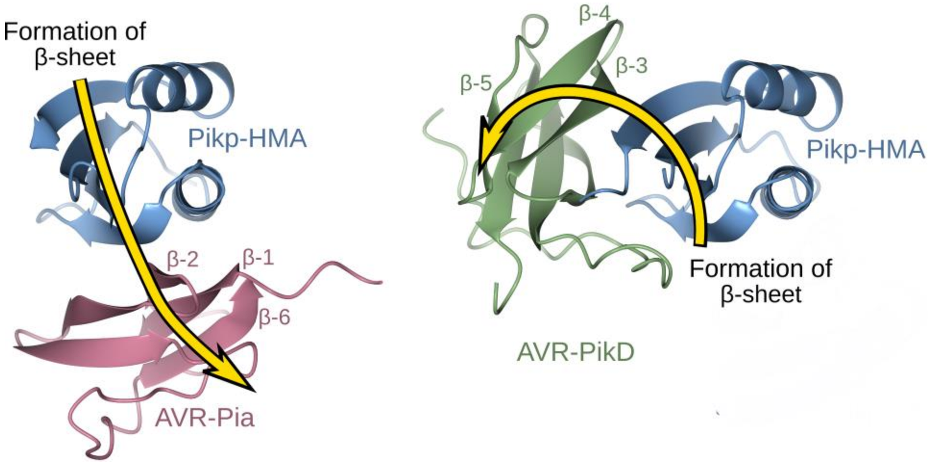

Figure S4. Schematic diagram highlighting the position of the continuous β-strands formed in the complexes between Pikp-HMA/AVR-Pia (left), and Pikp-HMA/AVR-PikD (right). Adapted from Fig. 4B.

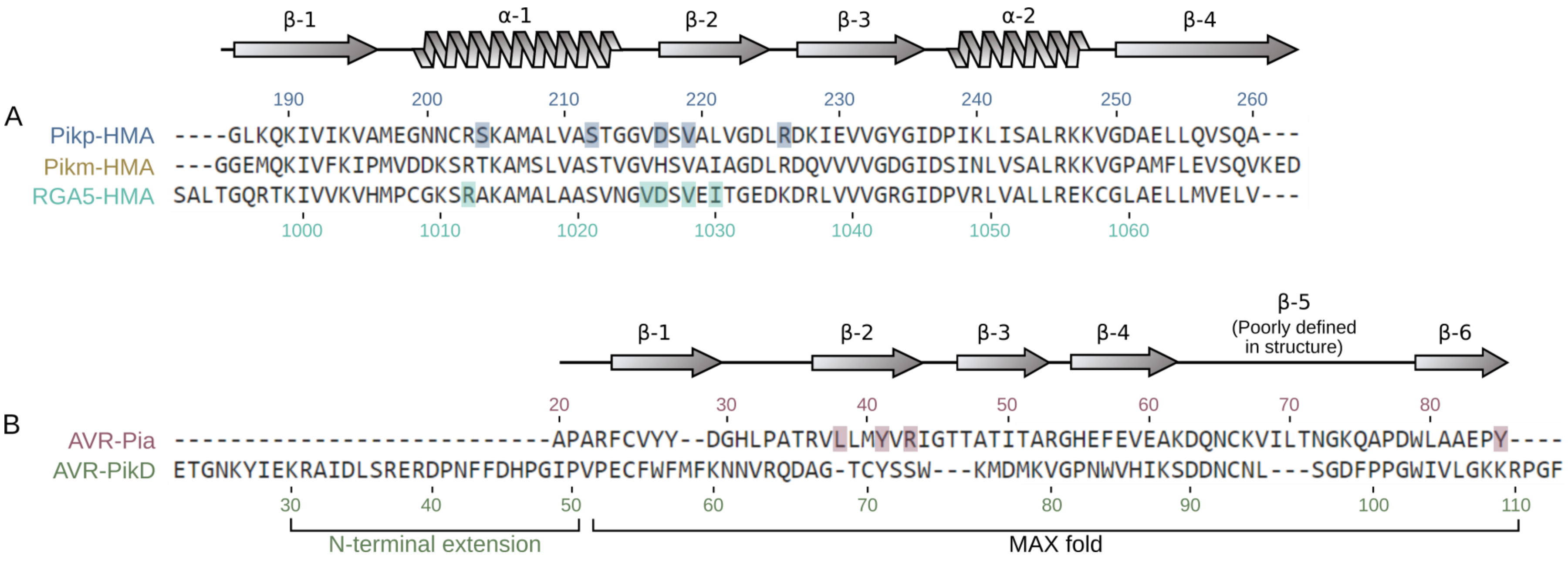

Figure S5. A) Sequence alignment of Pikp-HMA, Pikm-HMA and RGA5-HMA prepared with Clustal Omega (available at the European Bioinformatics Institute (EBI) website). This alignment only shows residues present in the PDB accessions 6Q76, 6FU9 (1) and 5ZNG (2) respectively. Residues are numbered for Pikp-HMA and RGA5-HMA. Key residues for the interaction with AVR-Pia or AVR1-CO39 (as described in the text and in (2)) are highlighted. B) Sequence alignment of AVR-Pia and AVR-PikD, with signal peptides removed. Areas comprising MAX fold (for both effectors) and N-terminal extension (for AVR-PikD only) are indicated. Key residues for the interaction with Pikp-HMA are highlighted. Secondary structure features from known structures are shown above the alignments in both panels.

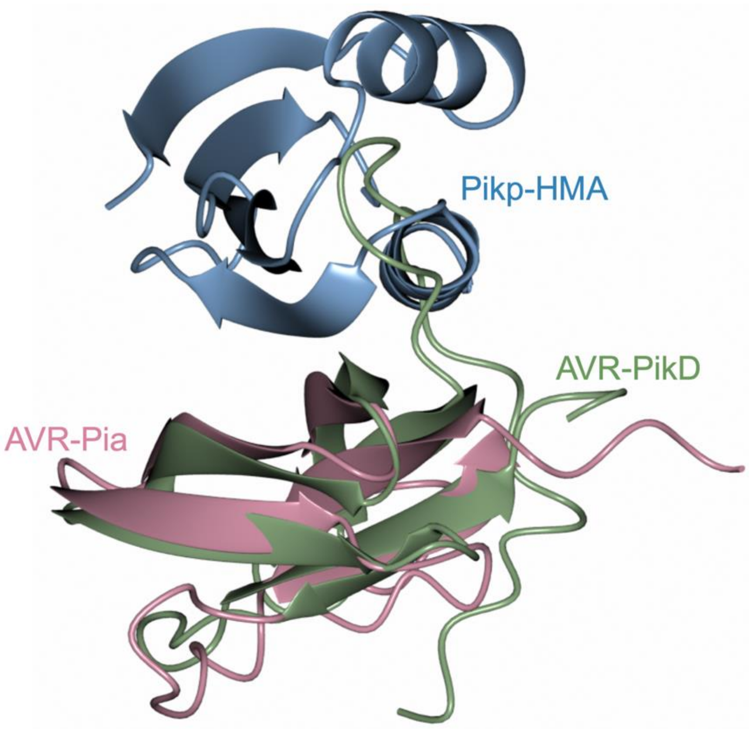

Figure S6. Schematic diagram showing a steric clash between Pikp-HMA and the N-terminal arm of AVR-PikD, if AVR-PikD were to bind Pikp-HMA at the Pikp-HMA/AVR-Pia interface. AVR-PikD has been overlaid on AVR-Pia, and all colours are as described in Fig 4.

REFERENCES

1. de la Concepcion, J. C., Franceschetti, M., Maqbool, A., Saitoh, H., Terauchi, R., Kamoun, S., and Banfield, M. J. (2018) Polymorphic residues in rice NLRs expand binding and response to effectors of the blast pathogen. *Nat Plants* **4**, 576-585
2. Guo, L., Cesari, S., de Guillen, K., Chalvon, V., Mammri, L., Ma, M., Meusnier, I., Bonnot, F., Padilla, A., Peng, Y. L., Liu, J., and Kroj, T. (2018) Specific recognition of two MAX effectors by integrated HMA domains in plant immune receptors involves distinct binding surfaces. *Proc. Natl. Acad. Sci. USA* **115**, 11637-11642
